# Effects of web-based interventions on cancer caregivers’ burden and quality of life: A systematic review and meta-analysis

**DOI:** 10.1017/S1478951525100370

**Published:** 2025-07-18

**Authors:** Myoungsuk Kim, Kelly R. Tan, Lorinda Adaire Coombs

**Affiliations:** 1 College of Nursing, Kangwon National University, Chuncheon-Si, Gangwon-Do, South Korea, Republic of Korea; 2 School of Nursing, Hillman Cancer Center, University of Pittsburgh, Pittsburgh, PA, USA; 3 School of Nursing, University of North Carolina-Chapel Hill, Lineberger Comprehensive Cancer Center, Chapel Hill, NC, USA

**Keywords:** Internet-based intervention, caregiver burden, quality of life, cancer, meta-analysis

## Abstract

**Objectives:**

As cancer incidence and survival rates rise, caregivers responsible for providing diverse support face increased burden and reduced quality of life (QoL). Although research on web-based interventions for this group is expanding, the impact of these interventions on caregiver burden and QoL remains unclear. This study aims to investigate the effects of web-based interventions on the caregiver burden and QoL of caregivers of patients with cancer.

**Methods:**

Searches were conducted in PubMed, Web of Science, Cochrane Library, CINAHL, Embase, and PsycINFO from database inception to 10 June 2024. Two reviewers independently assessed each study and extracted data. The risk-of-bias in the studies was evaluated using Cochrane’s Risk-of-Bias tool for randomized controlled trials. The intervention effects were calculated using R package Meta version 4.0.3, utilizing standardized mean differences (SMD; Hedge’s ĝ) to calculate pooled effect sizes with 95% confidence intervals (CI). Publication bias assessment and sensitivity analysis were conducted to ensure the robustness of the results.

**Results:**

We reviewed 13 randomized controlled trials; our analysis indicated a small effect size of web-based interventions on caregiver burden (SMD = −0.19, 95% CI: −0.36 to −0.01). However, sensitivity analysis concluded that the effect was very small or nearly absent. Additionally, there was no statistically significant effect on QoL (SMD = 0.15, 95% CI: −0.05 to 0.36).

**Significance of results:**

Web-based interventions did not significantly reduce caregiver burden or improve caregivers’ QoL. To improve caregiver burden and QoL in the future, comprehensive and tailored web-based interventions for this population are needed.

## Introduction

The population of individuals living with cancer requiring care continues to increase in tandem with rising cancer incidence rates and the development of novel cancer treatments that improve the length of survival. Each year, more than 2 million new cancer cases will be diagnosed in the United States (Siegel et al. 2024). Consequently, higher numbers of cancer survivors continue to live longer, with more complex care needs. These complex care needs are more frequently being cared for by caregivers at home and in the community (Kuluski et al. 2017).

Caregivers of individuals with cancer provide necessary support and care for individuals impacted by cancer. Caregivers assist or are responsible for a range of support, including, but not limited to, care coordination, complex medical care, day-to-day assistance with activities of daily living, and psychosocial support (Adashek and Subbiah 2020; National Alliance for Caregiving 2016). Individuals with cancer can require caregivers for several years and even longer after the initial cancer diagnosis. Depending upon their individual needs, caregivers may provide intermittent acute and intense periods of care or chronic daily care related to the cancer diagnosis and possible adverse treatment events (National Alliance for Caregiving 2016). It is estimated that cancer caregivers spend an average of 32.9 hours a week providing care over 1.9 years, but often longer (National Alliance for Caregiving 2016).

Care provision can often be demanding, leading to increased caregiver burden and reduced quality of life (QoL) (Girgis et al. 2013; Hopps et al. 2017). Caregiver burden is the perception of the cumulative strain experienced related to caregiving responsibilities and situations (Liu et al. 2020). The prevalence of caregiver burden among cancer caregivers is unknown, however a significant percentage experience high unmet needs, ranging from 40% to 55% (Kim et al. 2010) and psychological health-related issues (e.g., depression and anxiety) that are highly correlated with caregiving burden. Cancer caregiving is often characterized as highly burdensome because of the immense responsibility placed on caregivers to provide care at home without sufficient support or training. Cancer caregiving interventions ranging from skills training to psychotherapy have been developed and tested in response. A meta-analysis of 49 trials of cancer caregiving interventions demonstrated modest effects on quality of life and burden (Chow et al. 2023). Despite these modest effects, access to these interventions remains limited due to challenges engaging caregivers and caregivers lack of time to receive interventions. In response, over the last 10 years, a growing body of web-based interventions has focused on improving outcomes among cancer caregivers to improve reach and timing flexibility. Most developed web-based interventions typically follow a module-based approach to deliver psychoeducational materials, social support information, and online check-ins (Heynsbergh et al. 2018; Sun et al. 2019). These interventions were deemed highly feasible and acceptable (Heynsbergh et al. 2018; Sun et al. 2019). Still, the overall efficacy of these interventions on caregiving burden and QoL remains unclear (Shin et al. 2018).

A review of previous studies analyzing the effects of web-based interventions on caregiver burden and QoL among caregivers of cancer patients revealed that most studies have focused on systematic reviews (Kaltenbaugh et al. 2015; Lorca-Cabrera et al. 2020; Tang et al. 2014), with a limited number of meta-analyses available. Furthermore, previous meta-analyses have comprehensively examined various e-health interventions (Li et al. 2022), including web-based, telephone, and app-based interventions, making it difficult to determine the specific effects of web-based interventions. Additionally, the number of studies included in these meta-analyses was limited to 3 or 4, posing challenges in drawing definitive conclusions. Notably, to the best of the authors’ knowledge, no study has yet conducted a meta-analysis exclusively including randomized controlled trials (RCTs) to assess the effects of web-based interventions on caregiver burden and QoL. Therefore, a meta-analysis with stricter inclusion criteria, incorporating only high-quality RCTs, is needed to more precisely evaluate the effectiveness of web-based interventions.

This study aimed to determine the efficacy of web-based interventions in improving caregiver burden and overall QoL by conducting a systematic review and meta-analysis.

## Methods

This systematic review was performed per the Cochrane Handbook for Systematic Reviews of Interventions (Higgins et al. 2023). It reported the results in accordance with the Preferred Reporting Items for Systematic Reviews and Meta-Analyses guidelines (Page et al. 2021). This review was registered in PROSPERO (No. CRD42024549994).

### Data sources and search strategies

We comprehensively searched 6 databases, including PubMed, Web of Science, Cochrane Library, CINAHL, Embase, and PsycINFO, from inception to 10 June 2024. We used Medical Subject Headings (MeSH) terms, entry terms, Embase Subject Headings (Emtree), keywords, and free-text terms to search the databases. Supplemental Tables 1 to 6 present the search strategies utilized for each database. Moreover, we conducted a manual search by reviewing references of prior studies.

**Table 1.** Characteristics of the randomized controlled trial studies (*N* = 13)

First author (Year), Country	Participants	Sample size (R = E:C/A = E:C)	Mean age (SD) (E:C)	Female (%) (E:C)	Contents of web-intervention	Type of intervention	Duration of intervention	Instruments	Control group
Applebaum et al. (2018), USA	ICs of patients with any site or stage of cancer	42:42/22:23	48.3 (11.4): 51.9 (11.2)	90: 76	CCC Workshop: self-administered web-based program (meaning-centered psychotherapy)	Psychosocial intervention	14 weeks	1) Burden: CRA	Usual care
Applebaum et al. (2024), USA	Primary caregivers of cancer patients	75:75/49:36	Both group: 52.3 (14.0)	Both group: 66	Web-based CSS-CG (5 webcasts that offer education and experiential exercises related to the meaning of caregiving)	Psychosocial intervention	3 months	1) QoL: FACT-GP	Usual care
Bodschwinna et al. (2022), Germany	ICs of patients with cancer	30:30/30:29	47.67 (8.86): 46.66 (10.48)	76.7: 62.1	PartnerCARE: psycho-oncological online intervention (psychoeducation, CBT, supportive therapy, guided imagery techniques.)	Psychosocial intervention	2 months	1) Burden: BSFC-s	Waiting list
								2) QoL: VR − 12	
Boele et al. (2022), USA	Caregivers of patients with PMBT	80:40/51:35	53.29 (11.1): 52.00 (12.6)	63.8: 77.5	Nurse-led online needs-based support program (CBT and evidence-based psychoeducational materials)	Psychosocial intervention	8 weeks	1) Burden: CRA	Usual care
Chen et al. (2022), China	ICs of patients diagnosed with AC with metastasis	26:21/25:21	50.92 (13.838): 45.71 (11.845)	52.0: 52.38	WBDLRP (memory prompts: stimuli to evoke memories, review extraction: summarizing key experiences, mind space: emotional expression and wish sharing, e-legacy: video recording of the patient’s story)	Psychosocial intervention	4 weeks	1) Burden: ZBI	Usual care
DuBenske et al. (2014), USA	ICs of patients with advanced NSCLC	144:141/44:51	56.56 (12.86): 54.57 (12.21)	66.1: 70.5	CHESS (web-based lung cancer information, communication, and coaching system for caregivers)	Multicomponent intervention	6 months	1) Burden: CQOLC burden subscale	General internet
Duggleby et al. (2017), Canada	Male spouses of women with breast cancer	29:28/29:28	53.55 (11.19): 57.21 (9.29)	0: 0	Web-based psychosocial supportive intervention entitled MaTT (self-awareness, emotional management, caregiving role changes, expected health impacts, and key health information)	Multicomponent intervention	4 weeks	1) QoL: CQOL-C	Usual care
Köhle et al. (2021), Netherlands	Partner of a cancer patient/survivor	70:66/70:66	56.40 (11.15): 54.24 (11.03)	71.4: 69.7	Psychological web-based self-help intervention (psychoeducation, psychological and meditation exercises, practical tips, inspiring content)	Psychosocial intervention	3 months	1) Burden: CSI	Waiting list
Lambert et al. (2022), Canada	ICs of men with prostate cancer	16:17/10:13	NR	93.8: 93.8	TEMPO (enhancing confidence in self-management for psychosocial issues and developing self-regulation skills for physical activity adherence)	Self-management program	3 months	1) QoL: SF − 12	Usual care
Reblin et al. (2018), USA	Caregivers of patients with primary brain tumors	30:10/30:10	56.7 (119.9): 59.1 (10.0)	73.3: 80.0	eSNAP (practical support, information, communication, financial support, emotional support, and self-care)	Multicomponent intervention	6 weeks	1) Burden: ZBI	No intervention
Schuit et al. (2022), Netherlands	Partners of patients with incurable cancer	28:30/28:30	57 (10): 61 (14)	32: 30	eHealth self-management application (information and feedback, self-management advice, health care options)	Self-management program	3 months	1) Burden: CSI	Waiting list
								2) QoL: EQ − 5D	
Theiling (2016), Netherlands	Partners of cancer patients	39:53/39:53	57.4 (11.28): 53.1 (10.14)	74.4: 71.7	Web-based psychological self-help intervention (chronic stress management, dealing with worry and negative thoughts, communication, mindfulness exercises, meditation, practical information)	Psychosocial intervention	3 months	1) Burden: CSI	Waiting list
Xu et al. (2023), USA	Caregivers of cancer patients with newly created ostomies	16:7/12:5	48.06 (16.30): 38.00 (27.47)	68.75: 100	eHealth symptom and complication management program (health information, personalized feedback, self-management instructions, social support)	Self-management program	2 months	1) Burden: ZBI	Usual care

Note. A = Analyzed; AC = Advanced cancer; BSFC-s = Short Version of the Burden Scale for Family Caregivers; CBT = Cognitive Behavioral Therapy; CCC = The Care for the Cancer Caregiver; CHESS = Comprehensive Health Enhancement Support System; CRA = Caregiver Reaction Assessment; CQOLC = Caregiver Quality of Life Index–Cancer; CQOL-C Burden Subscale = Caregiver Quality of Life–Cancer Scale Burden Subscale; CSI = Caregiver Strain Index; CSS-CG = CancerSupportSource^TM^-Caregiver; EQ-5D = EuroQol-5D; eSNAP = Electronic Support Network Assessment Program; FACT-GP = Functional Assessment of Cancer Therapy – General Population; MaTT = Male Transition Toolkit; NSCLC = Non-small cell lung cancer; PMBT = Primary malignant brain tumor; R = Randomized; SF-12 = 12-item Short-Form Health Survey; TEMPO = Tailored, web-based, psychosocial and physical activity self-management program; VR-12 = Veterans RAND 12-Item Health Survey; WBDLRP = WeChat-based Dyadic Life Review Program; ZBI = Zarit Burden Interview.

### Inclusion and exclusion criteria

This review included studies that met the following criteria: (1) P (Population): unpaid and family caregivers of patients aged 18 years and older with a cancer diagnosis; (2) I (Intervention): web-based interventions (delivered through online platforms accessible via a web browser with an internet connection); (3) C (Comparison): usual care, general care, and waiting list; (4) O (Outcomes): outcome includes caregiver burden or QoL; (5) S (Study design): randomized controlled trials (RCTs).

Exclusion criteria included the following: (1) unpaid and family caregivers of cancer patients under 18 years old; (2) app-based intervention, phone call, text message, videoconferencing, and email; (3) studies combining interventions other than web-based interventions in the experimental group and implementing different interventions in the control group as well; (4) studies with only a protocol available; (5) studies with quasi-experimental designs.

### Study selection and data extraction

After removing duplicate references using EndNote 20 reference management software (Clarivate Analytics, Philadelphia, PA, USA), 2 reviewers (M.K. and L.A.C.) independently screened titles and abstracts according to the eligibility criteria. After uploading the full texts of the selected literature to the EndNote software, the 2 reviewers (M.K. and L.A.C.) reviewed the full texts to choose the studies for inclusion in the meta-analysis. Any discrepancies between the 2 authors during this process were resolved through discussion with a third reviewer (K.R.T.) until a consensus was reached.

Two reviewers (M.K. and L.A.C.) independently extracted data into a pre-designed structured template. Subsequently, a third reviewer (K.R.T.) verified the accuracy of the data extraction. We extracted general characteristics (first author, publication year), participant characteristics (sample size, mean age, female percentage), intervention characteristics (contents of web intervention, duration), outcome details (instruments), and control group from the included studies. Any discrepancies arising during the data extraction process were resolved through discussion among all authors.

### Risk-of-bias assessment

Two reviewers (M.K. & L.A.C.) conducted independent assessments of the risk-of-bias in the included studies using Cochrane’s Risk-of-Bias 2.0 tool (RoB2) for RCTs (Sterne et al. 2019). This tool requires the assessment of 5 domains and an overall risk-of-bias. Each domain was assessed as “low,” “some concerns,” or “high.” The overall risk-of-bias was evaluated as follows: if all 5 domains were rated “low,” it was assessed as “low risk-of-bias”; if “low” and “some concerns” were present together, it was rated “some concerns”; however, if any one of the 5 domains was rated “high,” it was evaluated as “high risk-of-bias.” The 5 domains comprised the randomization process, deviations from intended interventions, missing outcome data, measurement of the outcome, and selection of reported results. Inconsistencies arising during this process were resolved through discussions with a third reviewer (K.R.T.).

### Certainty of evidence assessment

The quality of evidence was evaluated using the GRADE approach (Schünemann et al. 2013), and the results were presented alongside GRADE ratings in Summary of Findings (SoF) tables (Higgins et al. 2023). The GRADE system evaluates 5 criteria (the risk-of-bias, inconsistency, indirectness, imprecision, and publication bias) on a scale of “high,” “moderate,” or “very low” quality (Ryan and Hill 2016). Additionally, the large magnitude of effect, dose–response, and impact of plausible confounding factors are considered to potentially upgrade the quality of evidence. The quality of evidence was independently assessed by 2 reviewers (M.K. & L.A.C.), and any discrepancies were resolved through discussion with a third reviewer (K.R.T.).

### Synthesis and statistical analysis

The meta-analysis used standardized mean differences (SMD; Hedge’s ĝ) to calculate pooled effect sizes with 95% confidence intervals (CI) due to using different rating scales, and a random effects model was employed (DerSimonian and Laird 2015). The overall effect size was interpreted according to Cohen’s suggestions as follows: small (0.2 ≤ SMD < 0.5), moderate (0.5 ≤ SMD < 0.8), or large (SMD ≥ 0.8) effects (Cohen 2013). The meta-analysis was conducted using the R package Meta version 4.0.3 (R Foundation for Statistical Computing, Vienna, Austria). The heterogeneity of each study was analyzed using *I*^2^ statistics (Higgins et al. 2023). *I*^2^ values of 0–40% might not be important, 30–60% may represent moderate heterogeneity, 50–90% substantial heterogeneity, and 75–100% considerable heterogeneity. Subgroup analysis was conducted for both the intervention duration and type to identify possible causes of heterogeneity, and meta-regression was performed for continuous variables such as mean age and sample size. Funnel plots and Egger’s tests were employed to detect publication bias in the included studies. We conducted a sensitivity analysis to evaluate the reliability of the pooled estimates.

## Results

### Study selection

**Figure 1.**
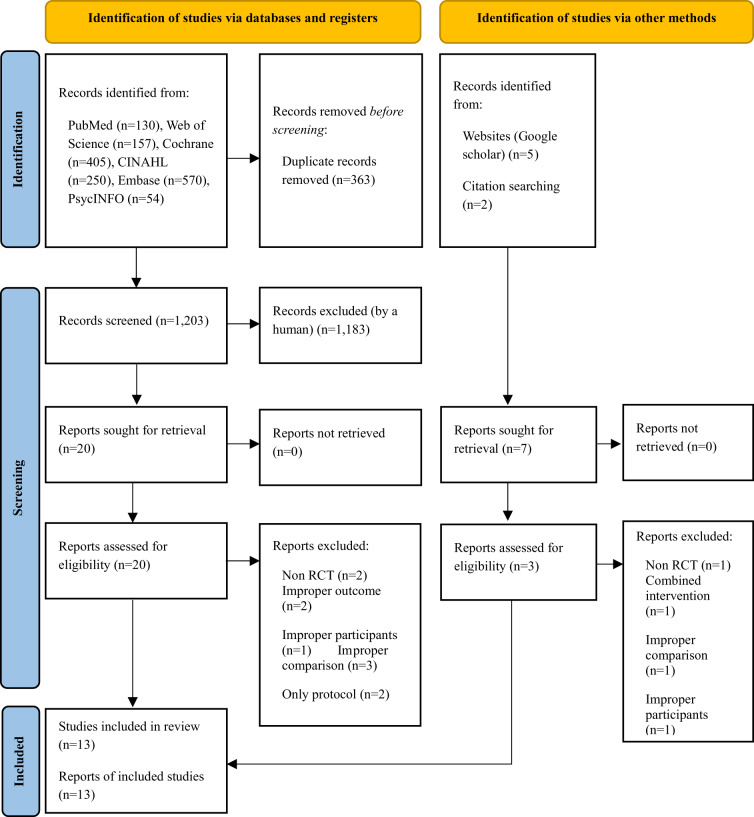
Flowchart summarizing the process of study selection.

From 6 databases, 1,566 articles were identified; after excluding duplicates, 1,203 articles remained. Among these, 1,183 articles were removed after reviewing only titles and abstracts, and 20 were included for full-text review. Among them, 10 articles were excluded for reasons such as non-RCT (*n* = 2), improper outcome (*n* = 2), improper participants (*n* = 1), improper comparison (*n* = 3), and only protocol (*n* = 2). Finally, 10 articles were included for the systematic review and meta-analysis. Seven articles were retrieved through the manual search of website and citations. After excluding 4 articles for reasons such as non-RCT (*n* = 1), combined intervention (*n* = 1), only protocol (*n* = 1) (Silveira et al. 2011), and improper participants (*n* = 1), 3 articles were included from the manual search. Finally, a total of 13 articles were included for the systematic review and meta-analysis (Figure. 1).

### Study characteristics

Table 1 presents the characteristics of the studies included in the meta-analysis. These studies were published between 2014 and 2024, with 8 out of 13 published after 2020. Six studies were conducted in the USA, 3 in the Netherlands, 2 in Canada, 1 in China, and 1 in Germany. The total sample sizes varied, ranging from 10 to 70 in the experimental group and 5 to 66 in the control group. The mean age was not reported in 1 study, while the average age across 12 studies was 52.6 years. The proportion of females in the experimental group ranged from 0% to 100%, with a mean of 64.5%.

In terms of intervention types, 7 studies utilized psychosocial interventions, 3 used self-management interventions, and 3 employed multicomponent interventions. Psychosocial interventions are aimed at enhancing an individual’s psychological and social well-being. They primarily include psychotherapy, emotional support, psychological counseling, and psychoeducational content. Multicomponent interventions integrate various elements such as psychological and emotional support, information provision, communication, and financial support. Self-management interventions are designed to help individuals take an active role in managing their own health and develop a sense of responsibility. They include self-management skills training, practical strategies, and behavior change facilitation. The intervention duration ranged from 4 weeks to 24 weeks, with 12 weeks being the most common across 5 studies.

Caregiver burden was measured using the Caregiver Strain Index (CSI) in 3 studies (Köhle et al. 2021; Schuit et al. 2022; Theiling 2016), Caregiver Reaction Assessment (CRA) in 2 studies (Applebaum et al. 2018; Boele et al. 2022), and the Zarit Caregiver Burden Scale in 2 studies (Reblin et al. 2018; Xu et al. 2023), among others such as the Short Version of the Burden Scale for Family Caregivers (BSFC-s) in 1 study (Bodschwinna et al. 2022), Zarit Caregiver Burden Interview in 1 study (Chen et al. 2022), and Caregiver Quality of Life Index–Cancer (CQOLC) burden subscale in 1 study (DuBenske et al. 2014).

QoL was measured using various instruments including Functional Assessment of Cancer Therapy–General Population (FACT-GP) (*n* = 1) (Applebaum et al. 2024), Veterans RAND 12-Item Health Survey (VR-12) (*n* = 1) (Bodschwinna et al. 2022), CQOL-C (*n* = 1) (Duggleby et al. 2017), SF-12 (12-item Short-Form Health Survey) (*n* = 1) (Lambert et al. 2022), and EQ-5D (EuroQol-5D) (*n* = 1) (Schuit et al. 2022). The most common intervention in the control group was usual care (*n* = 7).

### Risk-of-bias

**Figure 2.**
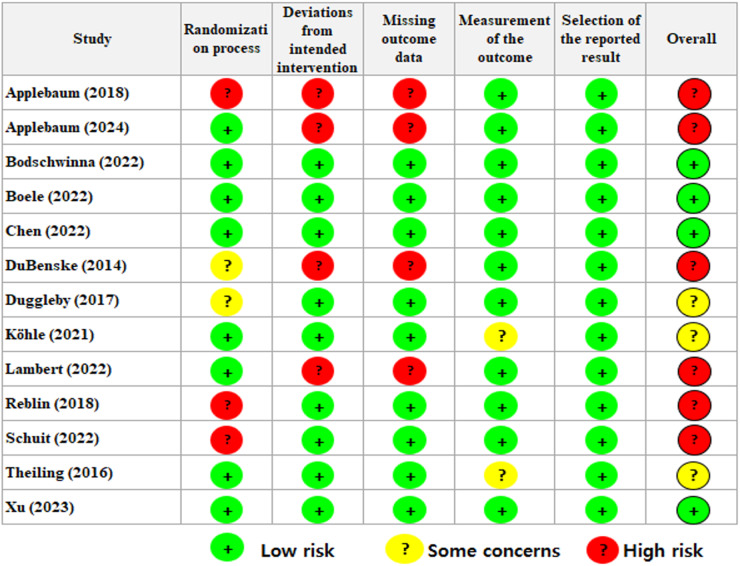
Risk-of-bias summary according to the revised cochrane risk-of-bias 2.0 tool for randomized trials.

Figure 2 presents the results of the risk-of-bias assessment. In the randomization process domain, 8 studies were rated “low risk,” while two studies were categorized as “some concerns” due to a lack of information regarding whether the allocation sequence – the method of randomly assigning participants to different groups – was concealed until the intervention assignment. Three studies were rated “high risk” because they did not randomize the allocation sequence and did not conceal it until intervention assignment. Regarding deviations from the intended interventions, 9 studies were rated “low risk.” In contrast, 4 studies were rated “high risk” because appropriate analysis, such as intention-to-treat, was not conducted. This could lead to bias from unintended deviations in the intervention, affecting the results.

In the missing outcome data domain, 9 studies were assessed as “low risk,” while 4 were rated “high risk” due to a significant amount of missing outcome data. In measuring the outcome, 11 studies were categorized as “low risk,” whereas 2 studies were classified as “some concerns” because assessors were aware of the interventions received by participants. In selecting the reported result, all studies were rated “low risk.” The overall risk-of-bias was evaluated as “low risk” for 4 studies, “some concerns” for 3, and “high risk” for 6.

### Effects of web-based interventions on caregiver burden

**Figure 3.**
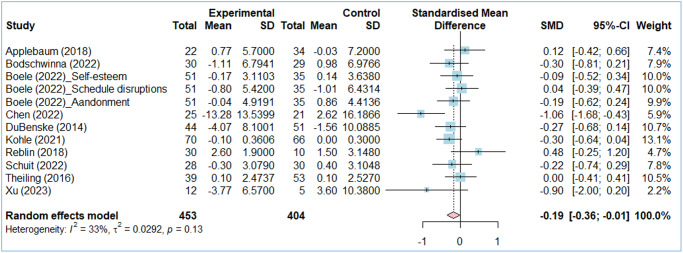
Forest plots: effect of web-based interventions on caregiver burden.

The effect of web-based interventions on cancer caregiver burden was analyzed using 12 comparisons derived from 10 studies. The pooled SMD for caregiver burden was −0.19 (95% CI: −0.36 to −0.01). Only 1 of the 12 comparisons showed a statistically significant effect, while the remaining 11 indicated no statistically significant effect. Heterogeneity was deemed “might not be important” (*I*^2^ = 33%) (Figure. 3).

### Effects of web-based interventions on QoL

**Figure 4.**
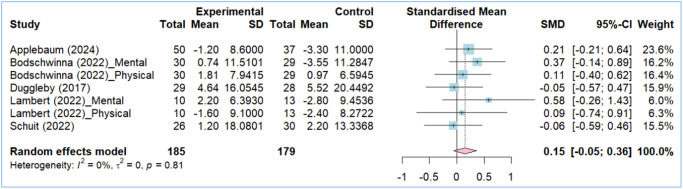
Forest plots: effect of web-based interventions on quality of life.

The effect of web-based interventions on caregivers’ QoL was analyzed using 7 comparisons from 5 studies. Web-based interventions showed no statistically significant impact on caregivers’ QoL (SMD = 0.15, 95% CI: −0.05 to 0.36). All individual studies also indicated no statistically significant effect (Figure. 4).

### Moderator analysis

Heterogeneity was low in caregiver burden; however, moderate analysis was conducted to identify potential factors that could influence it. First, subgroup analysis was performed on the intervention type and duration. Statistically significant differences between the groups were not observed in either subgroup, indicating no apparent causes of heterogeneity. Furthermore, the meta-regression results on mean age and sample size showed no statistically significant causes of heterogeneity for both variables (Table 2).

**Table 2.** Moderator analysis

						Between groups	
Moderators	Subgroup	*K*	SMD	95% CI	***I*^2^** (%)	Q_b_ (df)	*p_b_*
**Categorical moderators**	**Type of intervention**						
	Psychosocial intervention	8	−0.19	−0.41, 0.03	38.2	0.90 (2)	0.638
	Multicomponent intervention	2	−0.03	−0.50, 0.45	67.9		
	Self-management intervention	2	−0.38	−0.96, 0.18	16.2		
	**Duration of intervention**						
	≤ 6 weeks	4	−0.18	−0.51, 0.14	74.5	0.01 (1)	0.986
	≥ 2 months	8	−0.18	−0.40, 0.03	0		
**Continuous moderators**	**Variables**	***K***	**QM (df)**	**β**	**SE**	***p***	***R*^2^** (%)
	**Mean age**	12	2.54 (1)	0.038	0.024	0.110	21.43
	**Sample size**	12	0.09 (1)	0.001	0.003	0.757	0.0

Note. CI: Confidence interval; df: degree of freedom; *p*_b_; *p* between groups; Q_b_: Q between groups; QM: Q moderator.

### Publication bias

Publication bias was only analyzed in caregiver burden. First, a visual analysis was conducted using a funnel plot (Supplemental Figure 1). Subsequently, to provide more objective evidence, Egger’s regression analysis was performed, which revealed that it was not statistically significant (*t* = − 0.55, df = 10, *p* = 0.591). Thus, publication bias appears absent, and the meta-analysis results can be considered reliable.

### Sensitivity analysis

Sensitivity analysis was conducted to assess the robustness of the effect of web-based interventions on caregiver burden. Initially, using the Baujat plot (Supplemental Figure 2A) to identify the study that most influenced the results, we found that the study by Chen et al. (2022) had the greatest impact. The overall effect size was −0.19 (95% CI: −0.36 to −0.01), but after excluding the study by Chen et al. (2022), the effect size was −0.14 (95% CI: −0.28 to 0.00), indicating no significant effect. This suggests that, even before excluding the study by Chen et al. (2022), a very small effect size was present, which indicates the high reliability of our study results. Moreover, after excluding each of the 4 studies assessed as high risk-of-bias, 2 studies (Applebaum et al. 2018; Reblin et al. 2018) showed a slight increase in effect size from − 0.19 to −0.21, while the other 2 studies (DuBenske et al. 2014; Schuit et al. 2022) showed no effect (Supplemental Figure 2B). These results suggest that our meta-analysis provides robust findings.

### Certainty assessment using GRADE

The overall certainty of evidence for caregiver burden was rated “moderate.” This was due to the issue of the overall risk-of-bias, which led to a downward adjustment in the overall certainty of evidence. The overall certainty of evidence for QoL was assessed as “very low.” This was attributed to the risk-of-bias and inadequate sample size, resulting in it being rated “extremely serious” for imprecision. Additionally, the small number of studies included in the analysis meant that publication bias was not assessed (Supplemental Table 7).

## Discussion

### Interpretation of results and comparison with previous studies

Our study analyzed the effects of web-based interventions on caregiver burden and QoL among cancer caregivers. Web-based interventions are valuable and convenient, as they can be provided on a flexible schedule and accessed when in-person interventions are impossible (e.g., during COVID-19). Although systematic reviews on this topic have been conducted (Kaltenbaugh et al. 2015; Lorca-Cabrera et al. 2020; Tang et al. 2014), no meta-analysis has exclusively examined web-based interventions. Existing meta-analyses on e-health interventions (Li et al. 2022) include telephone- and mobile app-based interventions, making it difficult to isolate the independent effects of web-based interventions. Furthermore, the analysis of caregiver burden and QoL (Li et al. 2022) is based on a limited number of studies (3–4), which reduces the generalizability of the findings. Therefore, our study analyzed the effects of web-based interventions on caregiver burden and QoL in caregivers of patients with cancer, including only RCT studies.

The effect of web-based interventions on caregiver burden among cancer caregivers showed a statistically significant impact, although with a small effect size. However, excluding the study that had the most critical influence on effect size in the sensitivity analysis, web-based interventions resulted in nonexistent effects on caregiver burden. Therefore, limitations exist in concluding that web-based interventions impact caregiver burden. This finding is similar to that of a study involving caregivers of patients with cancer (Li et al. 2022), which showed no significant effect of e-health interventions on caregiver burden. However, e-health interventions encompass various health management strategies utilizing electronic technologies, including not only web-based interventions but also mobile applications, remote health monitoring, telemedicine, and other digital health approaches. Therefore, there are limitations in directly comparing the findings of this study with ours. Specifically, the study by Li et al. (2022) includes a total of 7 studies, of which only three are web-based interventions, limiting the ability to make a meaningful comparison with our study.

The very small effect size of web-based interventions on caregiver burden among cancer caregivers can be attributed to several factors. First, caregiver burden involves various attributes such as multifaceted strain, self-perception, and changes over time (Liu et al. 2020). It is influenced by factors such as long caregiving duration, caregiver retirement, lack of supplemental health insurance, older caregiver age, low-income level, high work requirements, high patient dependency, and high subjective stress (Akter et al. 2023; Karimi Moghaddam et al. 2023). Most of the studies included in our meta-analysis consist of psychosocial interventions, primarily aimed at providing psychosocial and emotional support, and improving coping strategies. However, since the caregiving burden of cancer patients is influenced by various factors, such interventions alone have limitations in effectively alleviating the actual burden of caregiving. Therefore, when developing web-based interventions to effectively reduce the burden on caregivers of cancer patients, a comprehensive approach is needed to address the various factors that contribute to caregiving burden. Specifically, beyond psychosocial support, interventions should include tailored support based on caregivers’ individual needs and circumstances, programs to promote caregivers’ physical health, stress management strategies, financial support systems, and systems that facilitate interaction with peers or professionals. Next, the studies included in our meta-analysis involved caregivers of cancer patients with diverse characteristics. Additionally, various factors that could influence caregiver burden, such as financial status, patient dependency, and caregiving duration, were not controlled for, which may have affected the results.

In terms of QoL, our meta-analysis showed that web-based interventions did not have a significant effect on improving caregivers’ QoL. This finding is somewhat inconsistent with the results of a previous study (Li et al. 2022), which showed a small effect size in enhancing caregivers’ QoL with e-health interventions. However, this previous study included overall interventions as e-health interventions, failing to demonstrate the effects of web-based interventions alone. Moreover, only 3 studies were included in the meta-analysis, which may have compromised the reliability of the results.

The web-based interventions may not have been effective in improving the QoL of family caregivers of cancer patients for several reasons. Factors influencing the QoL of family caregivers of patients with cancer include patient-related factors, caregiver-related factors, and environmental factors. Previous studies have shown that various factors, such as the patient’s functional decline, patient’s stress, caregiver’s low educational level, relationship between the patient and caregiver, caregiver stress, caregiver burden, and lack of social support, all impact the caregiver’s QoL (Liu et al. 2023, 2020; Maziyya et al. 2018; Yu et al. 2017). However, the studies included in our meta-analysis did not adequately control for several important factors affecting the QoL of cancer caregivers, which could explain why the interventions did not show a positive effect on caregivers’ QoL. Additionally, our meta-analysis included only 5 studies, which limits the ability to draw definitive conclusions about the effect on QoL. Furthermore, QoL should be assessed comprehensively across physical, psychological, social, and environmental dimensions. However, some studies only evaluated physical or mental aspects of QoL, which likely limited the overall assessment of caregivers’ QoL. Lastly, the included studies may have shown limited effects because they did not consider a comprehensive intervention that takes into account the various factors influencing the QoL of caregivers of cancer patients. To properly assess the effectiveness of web-based interventions aimed at improving the QoL of caregivers, future research should control for the various important factors that affect QoL and use a measurement tool that can evaluate QoL comprehensively. Furthermore, to develop an effective web-based intervention, it is essential to thoroughly understand the various factors affecting the QoL of cancer caregivers and develop strategies for incorporating these factors into the intervention program.

### Strengths and limitations

The strengths of our study are as follows: First, we included only RCTs in our analysis, ensuring the reliability of the effects of web-based interventions. Second, instead of conducting a meta-analysis of e-health interventions, we focused solely on the results of web-based studies, providing more precise outcomes. Third, to our knowledge, this is the first study to analyze the effects of web-based interventions on caregiver burden and QoL for caregivers with cancer.

Our study has several limitations. First, although we aimed to include high-quality RCTs for web-based interventions for caregivers of patients with cancer, such interventions are still limited for cancer caregivers. Second, most studies were conducted in the United States and Canada, with only 1 in Asia, making it difficult to generalize the results. Third, despite conducting a comprehensive search across multiple databases, gray literature was not included in this study; therefore, the results of this study should be interpreted with caution. Fourth, many studies included in the risk-of-bias assessment were categorized as “high risk,” which may reduce the reliability and validity of the study results, requiring careful interpretation. Lastly, in this study, moderator analysis was conducted based on assigned intervention categories. However, variations may exist within each category, and analyzing specific intervention approaches could be more meaningful. Due to the limited number of included studies, detailed subgroup analysis was not feasible. Future research should include a larger sample to account for these differences.

In our systematic review and meta-analysis, web-based interventions showed a negligible effect on the burden of caregivers of patients with cancer. However, further analysis concluded that the effect size may be small or nonexistent. Additionally, web-based interventions did not show a significant effect on improving the QoL of caregivers of patients with cancer. While our study has several limitations, these results demonstrate the need for caution when using web-based interventions to reduce caregiver burden and improve the QoL for caregivers of patients with cancer. Furthermore, developing comprehensive and tailored web-based intervention programs for caregivers of patients with cancer could be more effective in reducing their caregiver burden and improving their QoL.

## Supporting information

10.1017/S1478951525100370.sm001 Myoungsuk et al. supplementary material Myoungsuk et al. supplementary material

## Data Availability

The data supporting the findings of this study are available upon request.

## Supplementary material

The supplementary material for this article can be found at https://doi.org/10.1017/S1478951525100370.
