# Supplementary material for: Effects of web-based interventions on cancer caregivers’ burden and quality of life: A systematic review and meta-analysis
Source: Palliat Support Care. 2025 Jul 18;23:e134. doi: 10.1017/S1478951525100370 (PMC13166587; doi:10.1017/S1478951525100370)
Supplement: Myoungsuk et al. supplementary material [file S1478951525100370sup001.docx]

**Supplementary Materials**

**Supplemental Table 1.** PubMed

| **Search #** | **MeSH Terms and Key Words** | **Articles Revealed** |
| --- | --- | --- |
| #1 | "Caregivers"[MeSH Terms] OR "Spouse"[All Fields] OR "Family members"[All Fields] OR "friends"[All Fields] OR "Friending"[All Fields] OR "Friends"[MeSH Terms] OR "Friends"[All Fields] OR "Friend"[All Fields] OR "partner"[All Fields] OR "Primary caregivers"[All Fields] OR “I**nformal Caregivers**"[All Fields] | **2,402,121** |
| #2 | "Neoplasms"[MeSH Terms] OR "Cancer s"[All Fields] OR "Cancerated"[All Fields] OR "Canceration"[All Fields] OR "Cancerization"[All Fields] OR "Cancerized"[All Fields] OR "Cancerous"[All Fields] OR "Neoplasms"[MeSH Terms] OR "Neoplasms"[All Fields] OR "Cancer"[All Fields] | **5,085,368** |
| #3 | "Internet use"[MeSH Terms] OR "Internet based intervention"[MeSH Terms] OR "Telemedicine"[MeSH Terms] OR "Internet based"[All Fields] OR "Internet based"[All Fields] OR "Online based"[All Fields] OR "Online based"[All Fields] OR "Web based"[All Fields] OR "Web based"[All Fields] | **103,806** |
| #4 | "Caregiver Burden"[All Fields] OR "burden caregiver"[All Fields] OR "Caregiver Burdens"[All Fields] OR "Caregiver Stress"[All Fields] OR "Caregiver Stresses"[All Fields] OR "stress caregiver"[All Fields] OR "Care Burden"[All Fields] OR "Caregiving Stress"[All Fields] OR "Caregiving Stresses"[All Fields] OR "stress caregiving"[All Fields] OR "Caregiving Burnout"[All Fields] OR "burnout caregiving"[All Fields] OR ("Caregiver Burden"[MeSH Terms] OR ("caregiver"[All Fields] AND "burden"[All Fields]) OR "Caregiver Burden"[All Fields] OR ("caregiving"[All Fields] AND "burnouts"[All Fields])) OR "Care Giving Burden"[All Fields] OR ("Caregiver Burden"[MeSH Terms] OR ("caregiver"[All Fields] AND "burden"[All Fields]) OR "Caregiver Burden"[All Fields] OR ("burden"[All Fields] AND "care"[All Fields] AND "giving"[All Fields])) OR "burnout caregiver"[All Fields] OR "Caregiver Burnout"[All Fields] OR "Caregiver Exhaustion"[All Fields] OR ("Caregiver Burden"[MeSH Terms] OR ("caregiver"[All Fields] AND "burden"[All Fields]) OR "Caregiver Burden"[All Fields] OR ("exhaustion"[All Fields] AND "caregiver"[All Fields])) OR "Life Quality"[All Fields] OR "health related quality of life"[All Fields] OR "health related quality of life"[All Fields] OR "HRQOL"[All Fields] OR "Psychological Well-Being"[All Fields] OR "QOL"[All Fields] | **150,157** |
| #5 | "Randomized Controlled Trial"[All Fields] OR "Randomised Controlled Trial"[All Fields] OR "Randomized"[All Fields] OR "Randomised"[All Fields] OR "Randomization"[All Fields] OR "Randomisation"[All Fields] | **1,133,916** |
| #6 | #1 AND #2 AND #3 AND #4 AND #5 | **130** |

**Supplemental Table 2.** Web of Science

| **Search #** | **MeSH Terms and Key Words** | **Articles Revealed** |
| --- | --- | --- |
| #1 | **ALL=("Caregiver" OR "Carers" OR "Carer" OR "Care Givers" OR "Care Giver" OR "Spouse Caregivers" OR "Caregiver, Spouse" OR "Caregivers, Spouse" OR "Spouse Caregiver" OR "Family Caregivers" OR "Caregiver, Family" OR "Caregivers, Family" OR "Family Caregiver" OR "Informal Caregivers" OR "Caregiver, Informal" OR "Caregivers, Informal" OR "Informal Caregiver" OR “Spouse” OR "Family members" OR “Friends” OR “Relatives” OR “Primary”****)** | [**2,586,473**](https://www-webofscience-com.libproxy.kangwon.ac.kr/wos/woscc/summary/ae7c02ab-57f1-459f-8761-c95a98cf2e23-e2ae7e79/relevance/1) |
| #2 | **ALL=("Tumor" OR "Neoplasm" OR "Tumors" OR "Neoplasia" OR "Neoplasias" OR "Cancer" OR "Cancers" OR "Malignant Neoplasm" OR "Malignancy" OR "Malignancies" OR "Malignant Neoplasms" OR "Neoplasm, Malignant" OR "Neoplasms, Malignant" OR "Benign Neoplasms" OR "Benign Neoplasm" OR "Neoplasms, Benign" OR "Neoplasm, Benign")** | [**4,002,283**](https://www-webofscience-com.libproxy.kangwon.ac.kr/wos/woscc/summary/1a1f9c02-5243-4a59-89e9-3f2d9783d1a9-e2ae80f6/relevance/1) |
| #3 | **ALL=("Internet Uses" OR "Web Usage" OR "Web Use" OR "Internet Usage" OR "Internet Based Intervention" OR "Internet-Based Interventions" OR "Web-based Intervention" OR "Web-based Interventions" OR "Online Intervention" OR "Online Interventions" OR "Internet Intervention" OR "Internet Interventions" OR "Tele-Referral" OR "Tele Referral" OR "Tele-Referrals" OR "Virtual Medicine" OR "Tele-Intensive Care" OR "Tele Intensive Care" OR "Mobile Health" OR "mHealth" OR "Telehealth" OR "eHealth" OR "Internet based" OR "Internet-based" OR "Online based" OR "Online-based" OR "Web based" OR "Web-based")** | [**164,590**](https://www-webofscience-com.libproxy.kangwon.ac.kr/wos/woscc/summary/bfc063a1-e1ea-4ce0-9969-682aba5ab8df-e2ae8393/relevance/1) |
| #4 | **ALL=(**“Caregiver Burden” OR "Burden, Caregiver" OR "Caregiver Burdens" OR "Caregiver Stress" OR "Caregiver Stresses" OR "Stress, Caregiver" OR "Care Burden" OR "Caregiving Stress" OR "Caregiving Stresses" OR "Stress, Caregiving" OR "Caregiving Burnout" OR "Burnout, Caregiving" OR "Caregiving Burnouts" OR "Care Giving Burden" OR "Burden, Care Giving" OR "Burnout, Caregiver" OR "Caregiver Burnout" OR "Caregiver Exhaustion" OR "Exhaustion, Caregiver" OR "Life Quality" OR "Health-Related Quality Of Life" OR "Health Related Quality Of Life" OR "HRQOL" OR "Psychological Well-Being" OR "QOL") | [**157,560**](https://www-webofscience-com.libproxy.kangwon.ac.kr/wos/woscc/summary/0664a697-2b27-4b9b-ae3d-9db67f1d4bbb-e2aee12e/relevance/1) |
| #5 | ALL=("Randomized Controlled Trial" OR "Randomised Controlled Trial" OR "Randomized" OR "Randomised" OR "Randomization" OR "Randomisation") | [**997,111**](https://www-webofscience-com.libproxy.kangwon.ac.kr/wos/woscc/summary/bf9e0f8a-7ecc-4c9f-96e9-11b67a5b6914-e2ae8825/relevance/1) |
| #6 | #1 AND #2 AND #3 AND #4 AND #5 | [**157**](https://www-webofscience-com-ssl.libproxy.snu.ac.kr/wos/woscc/summary/784b6e7b-e8f0-42b3-b896-ee937cb22dde-aa0f9b7a/relevance/1) |

**Supplemental Table 3.** Cochrane Library

| **Search #** | **MeSH Terms and Key Words** | **Articles Revealed** |
| --- | --- | --- |
| #1 | MeSH descriptor: [Caregivers] explode all trees OR "Caregiver" OR "Carers" OR "Carer" OR "Care Givers" OR "Care Giver" OR "Spouse Caregivers" OR "Caregiver, Spouse" OR "Caregivers, Spouse" OR "Spouse Caregiver" OR "Family Caregivers" OR "Caregiver, Family" OR "Caregivers, Family" OR "Family Caregiver" OR "Informal Caregivers" OR "Caregiver, Informal" OR "Caregivers, Informal" OR "Informal Caregiver" OR “Spouse” OR "Family members" OR “Friends” OR “Relatives” OR “Primary” | **613,080** |
| #2 | MeSH descriptor: [Neoplasms] explode all trees OR "Tumor" OR "Neoplasm" OR "Tumors" OR "Neoplasia" OR "Neoplasias" OR "Cancer" OR "Cancers" OR "Malignant Neoplasm" OR "Malignancy" OR "Malignancies" OR "Malignant Neoplasms" OR "Benign Neoplasms" OR "Benign Neoplasm" | **289,660** |
| #3 | MeSH descriptor: [Internet Use] explode all trees OR MeSH descriptor: [Internet-Based Intervention] explode all trees OR MeSH descriptor: [Telemedicine] explode all trees OR "Internet Uses" OR "Web Usage" OR "Web Use" OR "Internet Usage" OR "Internet Based Intervention" OR "Internet-Based Interventions" OR "Web-based Intervention" OR "Web-based Interventions" OR "Online Intervention" OR "Online Interventions" OR "Internet Intervention" OR "Internet Interventions" OR "Tele-Referral" OR "Tele Referral" OR "Tele-Referrals" OR "Virtual Medicine" OR "Tele-Intensive Care" OR "Tele Intensive Care" OR "Mobile Health" OR "mHealth" OR "Telehealth" OR "eHealth" OR "Internet based" OR "Internet-based" OR "Online based" OR "Online-based" OR "Web based" OR "Web-based" | **28,324** |
| #4 | MeSH descriptor: [Caregiver Burden] explode all trees OR MeSH descriptor: [Quality of Life] explode all trees OR “Caregiver Burden” OR "Burden, Caregiver" OR "Caregiver Burdens" OR "Caregiver Stress" OR "Caregiver Stresses" OR "Stress, Caregiver" OR "Care Burden" OR "Caregiving Stress" OR "Caregiving Stresses" OR "Stress, Caregiving" OR "Caregiving Burnout" OR "Burnout, Caregiving" OR "Caregiving Burnouts" OR "Care Giving Burden" OR "Burden, Care Giving" OR "Burnout, Caregiver" OR "Caregiver Burnout" OR "Caregiver Exhaustion" OR "Exhaustion, Caregiver" OR "Life Quality" OR "Health-Related Quality Of Life" OR "Health Related Quality Of Life" OR "HRQOL" OR "Psychological Well-Being" OR "QOL" | **90,356** |
| #5 | MeSH descriptor: [Randomized Controlled Trial] explode all trees OR Randomized Controlled Trial OR Randomized OR Randomised OR Randomization OR Randomisation | **1,271,484** |
| #6 | #1 AND #2 AND #3 AND #4 AND #5 | **405** |

**Supplemental Table 4.** CINAHL

| **Search #** | **Subject Headings (MH) and Key Words** | **Articles Revealed** |
| --- | --- | --- |
| S1 | (MH "Caregivers") OR TX ( "Caregiver" OR "Carers" OR "Carer" OR "Care Givers" OR "Care Giver" OR "Spouse Caregivers" OR "Caregiver, Spouse" OR "Caregivers, Spouse" OR "Spouse Caregiver" OR "Family Caregivers" OR "Caregiver, Family" OR "Caregivers, Family" OR "Family Caregiver" OR "Informal Caregivers" OR "Caregiver, Informal" OR "Caregivers, Informal" OR "Informal Caregiver" OR “Spouse” OR "Family members" OR “Friends” OR “Relatives” OR “Primary” ) | 1,063,474 |
| S2 | (MM "Cancer Patients") OR (MH "Neoplasms") OR (MH "Cancer Survivors") OR TX ("Tumor" OR "Neoplasm" OR "Tumors" OR "Neoplasia" OR "Neoplasias" OR "Cancer" OR "Cancers" OR "Malignant Neoplasm" OR "Malignancy" OR "Malignancies" OR "Malignant Neoplasms" OR "Neoplasm, Malignant" OR "Neoplasms, Malignant" OR "Benign Neoplasms" OR "Benign Neoplasm" OR "Neoplasms, Benign" OR "Neoplasm, Benign" OR "Cancer”) | 1,035,589 |
| S3 | (MH "World Wide Web+") OR (MH "Internet+") OR (MH "World Wide Web+") OR (MH "Internet+") OR (MH "Telemedicine+") OR (MH "Telehealth+") OR TX **("Internet Uses" OR "Web Usage" OR "Web Use" OR "Internet Usage" OR "Internet Based Intervention" OR "Internet-Based Interventions" OR "Web-based Intervention" OR "Web-based Interventions" OR "Online Intervention" OR "Online Interventions" OR "Internet Intervention" OR "Internet Interventions" OR "Tele-Referral" OR "Tele Referral" OR "Tele-Referrals" OR "Virtual Medicine" OR "Tele-Intensive Care" OR "Tele Intensive Care" OR "Mobile Health" OR "mHealth" OR "Telehealth" OR "eHealth" OR "Internet based" OR "Internet-based" OR "Online based" OR "Online-based" OR "Web based" OR "Web-based")** | 253,868 |
| S4 | (MH "Quality of Life") OR (MH "Caregiver Burden") OR TX ( “Caregiver Burden” OR "Burden, Caregiver" OR "Caregiver Burdens" OR "Caregiver Stress" OR "Caregiver Stresses" OR "Stress, Caregiver" OR "Care Burden" OR "Caregiving Stress" OR "Caregiving Stresses" OR "Stress, Caregiving" OR "Caregiving Burnout" OR "Burnout, Caregiving" OR "Caregiving Burnouts" OR "Care Giving Burden" OR "Burden, Care Giving" OR "Burnout, Caregiver" OR "Caregiver Burnout" OR "Caregiver Exhaustion" OR "Exhaustion, Caregiver" OR "Life Quality" OR "Health-Related Quality Of Life" OR "Health Related Quality Of Life" OR "HRQOL" OR "Psychological Well-Being" OR "QOL" ) | 246,081 |
| S5 | (PT randomized controlled trials) OR TX ("Randomized Controlled Trial" OR "Randomised Controlled Trial" OR "Randomized" OR "Randomised" OR "Randomization" OR "Randomisation") | 508,049 |
| S6 | S1 AND S2 AND S3 AND S4 | 250 |

**Supplemental Table 5.** Embase

| **Search #** | **Emtree Terms and Key Words** | **Articles Revealed** |
| --- | --- | --- |
| #1 | 'caregiver'/exp OR 'caregiver' OR 'carers' OR 'carer' OR 'care givers' OR 'care giver' OR 'spouse caregivers' OR 'caregiver, spouse' OR 'caregivers, spouse' OR 'spouse caregiver' OR 'family caregivers' OR 'caregiver, family' OR 'caregivers, family' OR 'family caregiver' OR 'informal caregivers' OR 'caregiver, informal' OR 'caregivers, informal' OR 'informal caregiver' OR 'spouse' OR 'family members' OR 'friends' OR 'relatives' OR 'primary' | **3,448,163** |
| #2 | 'malignant neoplasm'/exp OR 'tumor' OR 'neoplasm' OR 'tumors' OR 'neoplasia' OR 'neoplasias' OR 'cancer' OR 'cancers' OR 'malignant neoplasm' OR 'malignancy' OR 'malignancies' OR 'malignant neoplasms' OR 'neoplasm, malignant' OR 'neoplasms, malignant' OR 'benign neoplasms' OR 'benign neoplasm' OR 'neoplasms, benign' OR 'neoplasm, benign' | **8,196,961** |
| #3 | 'internet use'/exp OR 'web-based intervention'/exp OR 'telemedicine'/exp OR 'internet uses' OR 'web usage' OR 'web use' OR 'internet usage' OR 'internet based intervention' OR 'internet-based interventions' OR 'web-based intervention' OR 'web-based interventions' OR 'online intervention' OR 'online interventions' OR 'internet intervention' OR 'internet interventions' OR 'tele-referral' OR 'tele referral' OR 'tele-referrals' OR 'virtual medicine' OR 'tele-intensive care' OR 'tele intensive care' OR 'mobile health' OR 'mhealth' OR 'telehealth' OR 'ehealth' OR 'internet based' OR 'internet-based' OR 'online based' OR 'online-based' OR 'web based' OR 'web-based' | **194,978** |
| #4 | 'caregiver burden'/exp OR 'quality of life'/exp OR 'caregiver burden' OR 'burden, caregiver' OR 'caregiver burdens' OR 'caregiver stress' OR 'caregiver stresses' OR 'stress, caregiver' OR 'care burden' OR 'caregiving stress' OR 'caregiving stresses' OR 'stress, caregiving' OR 'caregiving burnout' OR 'burnout, caregiving' OR 'caregiving burnouts' OR 'care giving burden' OR 'burden, care giving' OR 'burnout, caregiver' OR 'caregiver burnout' OR 'caregiver exhaustion' OR 'exhaustion, caregiver' OR 'life quality' OR 'health-related quality of life' OR 'health related quality of life' OR 'hrqol' OR 'psychological well-being' OR 'qol' | **764,847** |
| 5 | 'randomized controlled trial'/exp OR 'randomized controlled trial' OR 'randomised controlled trial' OR 'randomized' OR 'randomised' OR 'randomization' OR 'randomisation' | **1,615,068** |
| #6 | #1 and #2 and #3 and #4 and #5 | **570** |

**Supplemental Table 6.** PsycINFO

| **Search #** | **Main Subjects and Key Words** | **Articles Revealed** |
| --- | --- | --- |
| S1 | DE "Caregivers" OR TX ("Caregiver" OR "Carers" OR "Carer" OR "Care Givers" OR "Care Giver" OR "Spouse Caregivers" OR "Caregiver, Spouse" OR "Caregivers, Spouse" OR "Spouse Caregiver" OR "Family Caregivers" OR "Caregiver, Family" OR "Caregivers, Family" OR "Family Caregiver" OR "Informal Caregivers" OR "Caregiver, Informal" OR "Caregivers, Informal" OR "Informal Caregiver" OR “Spouse” OR "Family members" OR “Friends” OR “Relatives” OR “Primary”) | 419,940 |
| S2 | DE "Neoplasms" OR TX ("Tumor" OR "Neoplasm" OR "Tumors" OR "Neoplasia" OR "Neoplasias" OR "Cancer" OR "Cancers" OR "Malignant Neoplasm" OR "Malignancy" OR "Malignancies" OR "Malignant Neoplasms" OR "Neoplasm, Malignant" OR "Neoplasms, Malignant" OR "Benign Neoplasms" OR "Benign Neoplasm" OR "Neoplasms, Benign" OR "Neoplasm, Benign") | 109,616 |
| S3 | DE "Internet" OR DE "Internet Access" OR DE "Internet Usage" OR DE "Telemedicine" OR DE "Digital Interventions" OR TX ("Internet Uses" OR "Web Usage" OR "Web Use" OR "Internet Usage" OR "Internet Based Intervention" OR "Internet-Based Interventions" OR "Web-based Intervention" OR "Web-based Interventions" OR "Online Intervention" OR "Online Interventions" OR "Internet Intervention" OR "Internet Interventions" OR "Tele-Referral" OR "Tele Referral" OR "Tele-Referrals" OR "Virtual Medicine" OR "Tele-Intensive Care" OR "Tele Intensive Care" OR "Mobile Health" OR "mHealth" OR "Telehealth" OR "eHealth" OR "Internet based" OR "Internet-based" OR "Online based" OR "Online-based" OR "Web based" OR "Web-based") | 73,721 |
| S4 | DE "Caregiver Burden" OR DE "Burnout" OR DE "Quality of Life" OR DE "Health Related Quality of Life" OR DE "Well Being" OR “Caregiver Burden” OR "Burden, Caregiver" OR "Caregiver Burdens" OR "Caregiver Stress" OR "Caregiver Stresses" OR "Stress, Caregiver" OR "Care Burden" OR "Caregiving Stress" OR "Caregiving Stresses" OR "Stress, Caregiving" OR "Caregiving Burnout" OR "Burnout, Caregiving" OR "Caregiving Burnouts" OR "Care Giving Burden" OR "Burden, Care Giving" OR "Burnout, Caregiver" OR "Caregiver Burnout" OR "Caregiver Exhaustion" OR "Exhaustion, Caregiver" OR "Life Quality" OR "Health-Related Quality Of Life" OR "Health Related Quality Of Life" OR "HRQOL" OR "Psychological Well-Being" OR "QOL" | **145,871** |
| S5 | DE "Randomized Controlled Trials" OR DE "Randomized Clinical Trials" OR TX ( "Randomized Controlled Trial" OR "Randomised Controlled Trial" OR "Randomized" OR "Randomised" OR "Randomization" OR "Randomisation" ) | **115,181** |
| S6 | S1 AND S2 AND S3 AND S4 | 54 |

**Supplemental Table 7**. GRADE evidence profile and summary of findings table for the outcomes

| **Outcomes** | **Certainty assessment** | | | | | | | **Anticipated absolute effects**  **(95% CI)** |
| --- | --- | --- | --- | --- | --- | --- | --- | --- |
|  | **Participants (studies) Follow-up** | **Risk of bias** | **Inconsistency** | **Indirectness** | **Imprecision** | **Publication bias** | **Overall certainty of evidence** |  |
| **Caregiver burden** | 857 (12 RCTs) | serious**^a^** | not serious | not serious | not serious | none | ⨁⨁⨁◯ Moderate | SMD **-0.19**  (-0.36 to -0.01) |
| **Quality of life** | 364 (7 RCTs) | serious**^b^** | not serious | not serious | extremely serious**^c^** | publication bias strongly suspected**^d^** | ⨁◯◯◯ Very low | SMD **0.15** (-0.05 to 0.36) |
| CI: confidence interval; GRADE: grading of recommendations, assessment, development, and evaluation; RCT: randomized controlled trial; SMD: standardized mean difference | | | | | | | | |
| **GRADE Working Group grades of evidence** **High certainty:** we are very confident that the true effect lies close to that of the estimate of the effect. **Moderate certainty:** we are moderately confident in the effect estimate; the true effect is likely to be close to the estimate of the effect, but there is a possibility that it is substantially different. **Low certainty:** we have limited confidence in the effect estimate; the true effect may be substantially different from the estimate of the effect. **Very low certainty:** we have very little confidence in the effect estimate; the true effect is likely to be substantially different from the estimate of the effect. | | | | | | | | |
| **Explanation**  **a**. Overall risk of bias was evaluated as “high risk of bias” in 5 studies.  **b**. Overall risk of bias was evaluated as “high risk of bias” in 4 studies.  **c.** The sample size was not sufficient.  **d.** We were unable to analyze publication bias due to the small number of included studies, but we anticipate that publication bias is likely to be significant. | | | | | | | | |


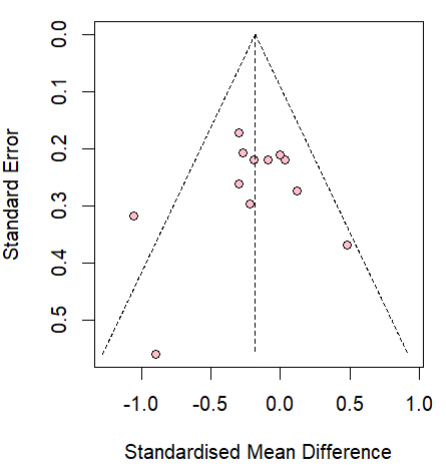


Supplemental Figure 1


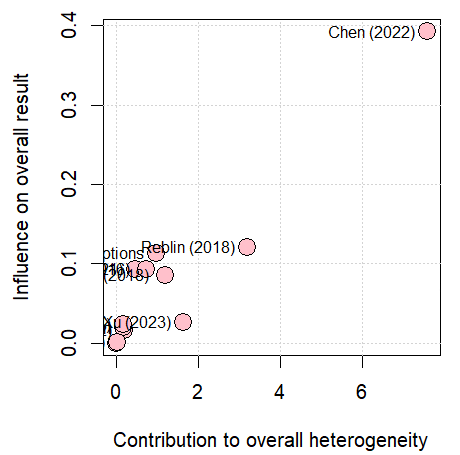

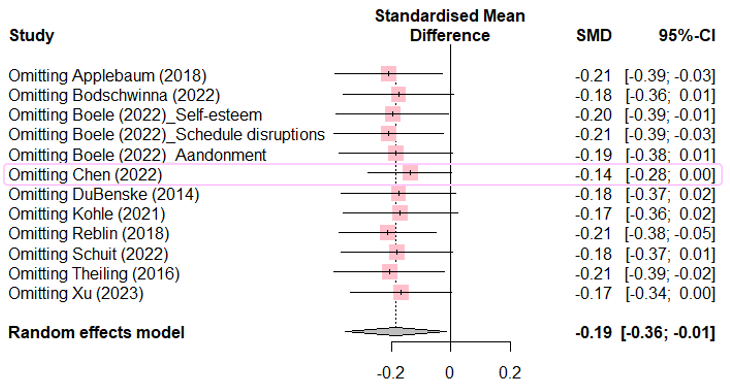


Supplemental Figure 2A Supplemental Figure 2B
